# Supplementary material for: Comparison of the clinical effects of computer-assisted and traditional techniques in bilateral total knee arthroplasty: A meta-analysis of randomized controlled trials
Source: PLoS One. 2020 Sep 25;15(9):e0239341. doi: 10.1371/journal.pone.0239341 (PMC7518627; doi:10.1371/journal.pone.0239341)
Supplement: Attachment — Submitted filename: Response to Reviewers.docx [file pone.0239341.s004.docx]

1.This article has been revised, yet, the English writing style is still required to be polished a little to make it more readable.

We read the full text carefully again and revised some places.

2. Fig 1: The details of reasons for full-text articles exclusion should be provided.

The exclusion criteria in this paper have been explained

3. P14: Only one study underwent staged bilateral TKAs within a period of 3 months. Which one? Please indicate.

We have already indicated it in this paper.

4. The conclusion is too long, and can be made to be shorten and concise.

We have simplified the conclusion part.

Thank you for your valuable comments！
